# Editorial: Treatment of psychopathological and neurocognitive disorders in genetic syndromes: In need of multidisciplinary phenotyping and treatment design

**DOI:** 10.3389/fpsyt.2022.1009376

**Published:** 2022-09-02

**Authors:** Jos Egger, Charlotte Egger, Kate Woodcock, Lotje De Witte, Marianne Van Den Bree, Linde Van Dongen, Ellen Wingbermühle, Tjitske Kleefstra

**Affiliations:** ^1^Donders Institute for Brain, Cognition and Behaviour, Radboud University, Nijmegen, Netherlands; ^2^Centre of Excellence for Neuropsychiatry, Vincent van Gogh Institute for Psychiatry, Venray, Netherlands; ^3^Stevig Specialized and Forensic Care for People With Intellectual Disabilities, Oostrum, Netherlands; ^4^Science Communication and Society, Institute of Biology, Leiden University, Leiden, Netherlands; ^5^School of Psychology, University of Birmingham, Birmingham, United Kingdom; ^6^Department of Psychiatry, Icahn School of Medicine at Mount Sinai, New York, NY, United States; ^7^Department of Psychiatry, Radboud University Medical Centre, Nijmegen, Netherlands; ^8^Division of Psychological Medicine and Clinical Neurosciences, MRC Centre for Neuropsychiatric Genetics and Genomics, Cardiff University, Cardiff, United Kingdom; ^9^Department of Genetics, Radboud University Medical Center, Nijmegen, Netherlands

**Keywords:** genetic neurodevelopmental disorder, contextual neuropsychology, perspective taking, psychopathological phenotype, etiology based assesment, interdisciplinary treatment

## Introduction

Similar to the figures in a coloring book, which are set at the moment of printing, the framework for a new person is set the moment a human egg is fertilized. The genes that we are born with contribute to our vulnerability for a wide range of possible phenotypes, but which of these phenotypes will subsequently develop, is greatly influenced by contextual factors from the physical world and social environments we live in, and our lifestyles (e.g., nutrition and exercise).

When looking at neurodevelopmental disorders with known genetic underpinnings, understanding the interaction between genes and context is vital for identification and support strategies. A fruitful way to achieve this would be through involvement of multiple disciplines, both in their additive capacities and in their ability to proceed from shared well-informed theoretical frameworks and clinical practice approaches. The inclusion of somatic, neuronal, cognitive and behavioral aspects, of rare genetic syndromes, and acquiring a more detailed understanding of altered brain development, new and fundamental insights can be gained, which can guide diagnosis and treatment decisions. In addition, a tailored approach to assessment and monitoring is necessary to understand how the contextual factors influence the (neuro)development of the individual patient under investigation. In the present Research Topic, nine studies have been brought together that illustrate ways to better assess the challenges that come with genetic neurodevelopmental disorders, to ameliorate symptoms, and in general to improve quality of life, not only of these individuals but also of their family members and other caregivers.

## Personalized healthcare

The phenotype of individuals with genetic neurodevelopmental disorders can differ considerably, as a consequence of their individual gene x environment developmental history. For example, the social abilities of caregivers have a substantial impact on the social development of a child. Because of this, personalized healthcare is important. Landlust et al. adopted a multidisciplinary method of assessment which takes the full developmental context into account and therefore allows more meaningful interpretations and predictions of behavior. Also, the Mobile-Health project by Heunis et al. aims to enhance personalized healthcare by using a self-report checklists to document the individual needs of patients and provide a toolkit with recommendations for support strategies.

## Early interventions

Since the context in which a child grows up can largely influence the development of the cognitive, social and behavioral symptoms, earlier interventions may have better developmental outcomes than later ones. Garg et al. found worse sleep behavior in infants with Neurofibromatosis type 1 compared to infants without a family history of neurodevelopmental difficulties. Interventions to promote sleep hygiene may therefore be an interesting early treatment option.

Personalized guidance on social behavior and social cognitive training may be another way in which early interventions can improve developmental outcomes and improve the quality of life for the affected individuals and their social network of family members and caretakers. Bouw et al. saw children between the age of 4–8 with Sex Chromosome Trisomies improve their facial emotion recognition skills drastically by using a neurocognitive training program. Their ability to identify standard facial communication of emotions improved to the extent that it was indistinguishable from non-disabled controls after completing the training. Social training can, however, also be beneficial to individuals with a genetic disorder of older ages. Dykens et al. developed an intervention to ameliorate social skill deficits in people aged 14–33 with Prader-Willi Syndrome. This intervention was seen to improve social cognition, motivation and communication skills of the affected persons and was linked to their reports having more friendships and reduced loneliness.

Early interventions may also have wider benefits, extending beyond the patient to their wider social network of family members and caretakers. Bos-Roubos et al. found a high prevalence of traumatic events and greater vulnerability to Posttraumatic Stress Disorder in family members of individuals with Prader-Willi Syndrome (PWS). The number and complexity of symptoms in individuals with PWS as well as the level of trauma symptom severity of their family members was positively associated with patient age. Thus, early interventions like those of Dykens et al. may not only improve the quality of life of the patients but also hold promise for the wider family.

## Biology in context

Even though genetic neurodevelopmental disorders often present with a highly variable phenotype (i.e., symptoms can differ significantly within genetic syndromes), comparing symptoms *between* syndromes may reveal information about their neurobiological mechanisms. For example, Lubbers et al. compared profiles of autism symptoms between individuals with different genetic syndromes and found similarities between sets of syndromes. These similarities may reflect similarities in the underlying neurobiology and provide a frame of reference, which can guide both research and treatment decisions. Similarly, Alfieri et al. found evidence that the differences in adaptive functioning on the socialization domain in children with Duplication 7 Syndrome or Williams-Beuren Syndrome may arise from general differences between the syndromes, including levels of cognitive function, a finding which provides insight into the developmental mechanisms of symptoms.

Understanding developmental mechanisms also implies the possibility of influencing them and can hence be important for designing new types of treatment. In recent years, studies have found that the composition of the gut microbiome is different in children with Autism Spectrum Disorder (ASD) compared to typically developing children. Whether these microbiome differences contribute to the behavioral symptoms remains to be understood. However, Lu et al. reviewed the literature on interventions to rebalance the gut microbiome and found reason to believe that probiotic treatment and microbiota transfer therapy may improve different behavioral symptoms of ASD.

As we have seen, genes and body, cognition and behavior as well as the environmental context are all interconnected and interact. The multiple disciplinary approach that was used in and across these studies is therefore vital for improved understanding of the phenotypic presentations and mechanisms of genetic neurodevelopmental disorders. Contextual parameters should furthermore also be taken into account by means of tailored assessments. Early interventions, like social trainings, can influence the course of development and therefore improve the quality of life of patients and their relatives and caregivers. Interpreting genetic disorders within the context of individual development, can be highly valuable for efforts to provide efficient and effective healthcare.

## Embodied embedded cognition pointing the way?

**Figure 1:**
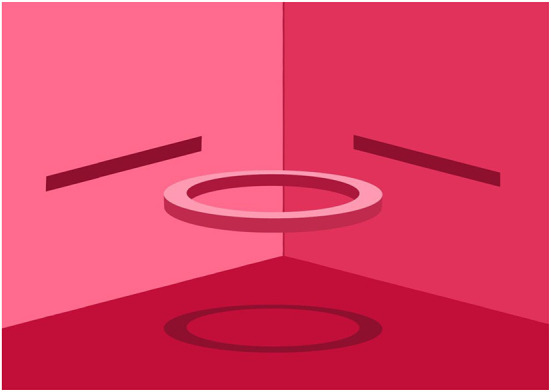
Different perspectives are needed: one eye cannot discern the ring.

In summary, the present Research Topic may teach us that the combined etiology-inspired and context-sensitive approach employed in an inter- and transdisciplinary manner is essential for understanding both the neurobiological and developmental mechanisms involved. As metaphorically depicted in Figure 1, this approach is probably beneficial for providing the optimal scope and perspectives for devising innovative treatment interventions.

## Author contributions

JE and CE: conceptualization and literature review and visualization. JE, CE, and EW: writing—original draft preparation. KW, LDe, MV, LV, and TK: writing—review and editing for important content. JE, EW, and TK: supervision. All authors have read and agreed to the final version of the manuscript.

## Conflict of interest

The authors declare that the research was conducted in the absence of any commercial or financial relationships that could be construed as a potential conflict of interest.

## Publisher's note

All claims expressed in this article are solely those of the authors and do not necessarily represent those of their affiliated organizations, or those of the publisher, the editors and the reviewers. Any product that may be evaluated in this article, or claim that may be made by its manufacturer, is not guaranteed or endorsed by the publisher.

